# Characterization of the Fecal and Mucosa-Associated Microbiota in Dogs with Chronic Inflammatory Enteropathy

**DOI:** 10.3390/ani13030326

**Published:** 2023-01-17

**Authors:** David Díaz-Regañón, Mercedes García-Sancho, Alejandra Villaescusa, Ángel Sainz, Beatriz Agulla, Mariana Reyes-Prieto, Antonio Rodríguez-Bertos, Fernando Rodríguez-Franco

**Affiliations:** 1 Department of Animal Medicine and Surgery, College of Veterinary Medicine, Complutense University of Madrid, Avda. Puerta de Hierro s/n, 28040 Madrid, Spain; 2 Department Medicina i Cirurgia Animals, Facultat de Veterinària, Universitat Autònoma de Barcelona, Bellaterra, 08193 Cerdanyola del Vàlles, Spain; 3 Sequencing and Bioinformatics Service, Foundation for the Promotion of Sanitary and Biomedical Research of the Valencian Community, 46020 Valencia, Spain; 4 VISAVET Health Surveillance Centre, Complutense University of Madrid, Avda. Puerta de Hierro s/n, 28040 Madrid, Spain

**Keywords:** dog, IBD, chronic enteropathy, gut microbiota, bacterial diversity, bacterial composition, 16S rRNA

## Abstract

**Simple Summary:**

Chronic inflammatory enteropathies are the most common cause of chronic vomiting and diarrhea in dogs. The pathogenesis of this disease is known to be multifactorial, where intestinal barrier dysfunction, immunological dysregulation and gut microbiota changes play a central role. Most sequencing studies assessing the intestinal microbiota in canine species have been made to evaluate fecal samples. Therefore, in this study, we aimed to characterize the intestinal bacterial microbiota from duodenal biopsies and fecal samples in dogs with inflammatory bowel disease at the diagnosis time and to compare it to healthy dogs. Our study results demonstrate that dogs with inflammatory bowel disease have significantly different gut microbiota when compared to healthy control dogs, but these changes are more obvious in the fecal microbiota rather than in the duodenal mucosal-associated one. Further investigations including functionality approaches targeting the gut microbiome at both levels are warranted.

**Abstract:**

Canine chronic inflammatory enteropathy implicates multifactorial pathogenesis where immunological dysregulation and gut microbiota changes have a central role. Most sequencing-based taxonomic studies have been focused on the fecal microbiota. However, the analysis of these samples does not provide complete information regarding the composition of the small intestine affected by this canine disease. Therefore, in this study, we aimed to characterize the intestinal bacterial microbiota in dogs with inflammatory bowel disease (IBD) (*n* = 34) by means of duodenal biopsies and fecal samples collected at the time of the diagnosis and to compare those to a group of healthy dogs (*n* = 12) using the 16S ribosomal RNA (16S rRNA) gene-targeted sequencing (Illumina MiSeq platform). Our study showed that IBD dogs presented differences in the fecal bacterial communities when compared with healthy dogs, with a lower relative abundance of Prevotellaceae (*p* = 0.005), *Prevotella* (*p* = 0.002), and *Prevotellaceae Ga6A1 group* (0.006); Erysipelotrichales (*p* = 0.019), *Candidatus* Stoquefichus (*p* < 0.001), Erysipelotrichaceae (*p* = 0.011), and *Allobaculum* (*p* = 0.003); *Lachnospiraceae NK4A136 group* (*p* = 0.015), *Sellimonas* (*p* = 0.042), Oscillospirales (*p* = 0.037), Oscillospiraceae *UCG–005* (*p* < 0.001), *Faecalibacterium* (*p* = 0.028), and *Fournierella* (*p* = 0.034); Acidaminococcales, Acidaminococcaceae, and *Phascolarctobacterium* (*p* = 0.001); Aeromonadales (*p* = 0.026), Succinivibrionaceae (*p* = 0.037), and *Succinivibrio* (*p* = 0.031). On the other hand, a higher relative abundance of Enterococcaceae (*Enterococcus*; *p* = 0.003), Streptococcaceae (*Streptococcus*, *p* = 0.021), Enterobacterales (*p* = 0.027), Enterobacteriaceae (*p* = 0.008), and *Escherichia–Shigella* (*p* = 0.011) was detected. Moreover, when evaluating α–diversity, the dogs with IBD showed lower diversity in terms of richness and abundance of species (observed species [*p* = 0.031] and Shannon index [*p* = 0.039]). Furthermore, fecal microbiota in dogs with IBD was significantly different from healthy dogs (*p* = 0.006). However, only a few taxa relative abundance shifts (lower Rubrobacteria, Rubrobacterales, Rubrobacteriaceae, and *Rubrobacter* [*p* = 0.002]; Cyanobacteria [*p =* 0.010], Vampirivibrionia, Obscuribacterales, and Obscuribacteraceae [*p* = 0.005]; Neisseriaceae [*p =* 0.004] and *Conchiformibius* [*p =* 0.003]) were observed when assessing duodenal-associated microbiota of dogs with IBD. Thus, even if the bowel inflammation mainly affects the small intestine in the IBD-affected dogs of the study, fecal specimens may constitute a better sample due not only to their easy availability but also in terms of searching for bacterial taxa as biomarkers for canine IBD. The use of different diets in the study can also have a partial influence on the microbiota composition. Future studies encompassing multi-omics approaches should evaluate the functionality in both levels to unravel the pathophysiology of canine IBD.

## 1. Introduction

Canine chronic inflammatory enteropathies (CIE) are a group of complexes, nonspecific gastrointestinal (GI) disorders that are diagnosed based on the presence of chronic GI signs (lasting 3 weeks or longer), the histopathologic evidence of intestinal mucosal inflammation, and the exclusion of other underlying causes [1,2,3].

These diseases are currently subclassified retrospectively based on the treatment response [3,4]. Thus, CIE is categorized as a food-responsive enteropathy (FRE) if the clinical signs significantly improve or resolve after a dietary trial with either a limited-ingredient novel protein or a hydrolyzed protein diet [5]. Another subgroup of dogs shows a marked improvement or resolution of their clinical signs after an antibiotic trial and has been classified as antibiotic-responsive enteropathy (ARE), recently proposed as “idiopathic intestinal dysbiosis” [4]. However, the high rate of relapsing cases after discontinuing the treatment, the profound effects on the intestinal microbiome, and antibiotic resistance as a major global concern, make this empirical antimicrobial approach controversial [6,7]. Disorders that require treatment with glucocorticoids or other immunosuppressive drugs are defined as steroid-responsive enteropathy (SRE) or immunosuppressant-responsive enteropathy (IRE) which diagnosis is achieved after carrying out an exclusion protocol and often is referred to as canine idiopathic inflammatory bowel disease (IBD). Finally, some dogs have no adequate response to immunosuppressant treatments, so the enteropathy is categorized as non-responsive (NRE) [3,4].

The etiopathogenesis of canine CIE is still relatively unknown but involves loss of tolerance to diet and microbial components that cause an aberrant immune response in genetically susceptible individuals, affecting dogs of any sex, age, or breed [4,8]. The factors that seem to play a significant role in the development of CIE include genetics, diet components, the integrity of the intestinal barrier, the intestinal immune response, and the GI microbiota [9,10,11,12].

The GI microbiota is a complex population of microorganisms, including not only bacteria but also archaea, fungi, protozoa, and viruses, which has a determinant role on the health status of the canine host [13,14,15,16]. Bacterial microbiota is the most studied and has been linked to key physiologic processes including nutrient absorption, vitamin synthesis (vit. K and complex B), energy metabolism, immune regulation, and maintenance of the GI barrier [16,17]. Thus, intestinal dysbiosis is associated with mucosal inflammation and GI dysfunction in dogs with CIE [4,18,19]. These microbial imbalances are characterized by marked shifts in the bacterial composition, reduced species diversity, and changes in the relative proportion of selected microbial members, as well as alterations in their metabolic activity, when compared to healthy dogs [4,19,20].

Most sequencing-based taxonomical studies have been focused on the fecal microbiota due to the ease of this type of sampling in the veterinary clinical setting [21]. These studies showed a reduction in the relative abundances of bacteria belonging to Bacteroidetes and Firmicutes phyla and *Fusobacterium* spp., and an increased abundance of Proteobacteria [19,22,23,24,25]. However, the analysis of these samples does not provide complete information regarding the composition of the small intestine microbiota [21]. Some studies have shown more consistent changes in mucosal microbiota rather than fecal microbiota in human GI disorders, inspiring researchers in this field to focus on mucosal microbiota rather than fecal microbiota [26].

Therefore, in this study, we aimed to characterize the intestinal bacterial microbiota in dogs with IBD by means of duodenal biopsies and fecal samples collected at the time of diagnosis and to compare those to samples collected from healthy dogs.

## 2. Materials and Methods

### 2.1. Dogs

A total of forty-six privately owned dogs attending the Complutense Veterinary Medicine Teaching Hospital (CVMTH) were prospectively enrolled between 2018 and 2021. All pet owners were written and orally informed by a veterinarian of the CVMTH regarding the potential risk of the procedure prior to inclusion in the study. Written informed consent was obtained from all owners of the included dogs. Data regarding signalment and epidemiological features were recorded. All procedures and protocols were approved by the Animal Research Committee of the CVMTH, the Complutense University of Madrid, and the Community of Madrid (PROEX 175/18).

#### 2.1.1. Healthy Control (HC) Dogs

Twelve dogs of different ages, breeds, and sexes were included in this group. All of them lived in diverse home environments, were fed different commercial maintenance diets, and were presented to the CVMTH for elective or routine consultations (e.g., orchiectomy or ovariohysterectomy). All dogs were judged to be healthy based on a normal physical examination, absence of abnormalities in the complete blood count and basic biochemistry, and negative serology (IFA) results to canine leishmaniosis and monocytic ehrlichiosis, the most prevalent canine vector-borne diseases in our geographical area [27]. In addition, an intestinal inflammatory process was ruled out based on the histopathological study of the GI biopsies. None of the dogs presented clinical GI signs within the six months before sampling, nor received antibiotics, probiotics or prebiotics, or other drug therapy at least one month prior to sampling. Any dog with a concurrent disease was excluded.

#### 2.1.2. IBD Dogs

A total of thirty-four patients diagnosed with IBD were included in the study. The diagnosis was made by a clinician of the Gastroenterology and Endoscopy team of the CVMTH based on the World Small Animal Veterinary Association (WSAVA) criteria [1]. To rule out other causes of GI inflammation or systemic diseases an exclusion diagnosis protocol was carried out. This protocol included a complete physical examination, complete blood count, serum biochemistry panel, IFA test against *Leishmania infantum* and *Ehrlichia canis*, direct (wet mount) and indirect (modified Telemann and merthiolate iodine formaldehyde) fecal examination for nematode and protozoan parasites detection, TLI (trypsin-like immunoreactivity), resting cortisol/ACTH stimulation test, and diagnostic imaging (abdominal ultrasound and/or radiographs). Furthermore, all dogs were fed a hydrolyzed protein-based diet for at least 3 weeks to discard an FRE [28,29,30]. Subsequently, they underwent an upper GI endoscopy and biopsy samples were taken to confirm the inflammatory process. All cases were scored for severity according to the clinical IBD activity index (CIBDAI) and the clinical canine chronic enteropathy activity index (CCECAI) [28,31]. Exclusion criteria included other causes for chronic GI signs besides IBD, treatment with antimicrobials, anti-inflammatory drugs, or both within one month before sample collection. Moreover, dogs with signs of large bowel GI disorders that needed bowel preparation for colonoscopy were ruled out of the study.

### 2.2. Sample Collection, Upper GI Endoscopy, and Histopathological Evaluation

Prior to the GI endoscopy, fresh rectal feces were collected from all dogs (*n* = 46). Sterile swabs (Deltalab, Barcelona, Spain) were inserted into the rectum and swept in a circular motion ensuring an adequate amount of sample. Subsequently, upper GI endoscopy was performed in all dogs. Video endoscopes of variable lengths were used according to the size of the dog (Fujinon [Europe] Gmblt, Willich, Germany). Food and water were removed from dogs 24 and 12 h prior to the endoscopy, respectively, for a proper evaluation. Macroscopic GI lesions were evaluated during the endoscopic procedure using the WSAVA endoscopic guidelines [1], and the endoscopic activity scores described by Slovak and coworkers [32]. All the alterations observed were filled out in proper forms by experienced endoscopists of the CVMTH. Six to seven biopsy specimens were taken from gastric and duodenal mucosa and preserved in 10% neutral-buffered formaldehyde for 48 h before being embedded in paraffin and subsequently prepared for histopathological evaluation. The endoscopic biopsies were examined histologically by an experienced pathologist. The histological alterations were scored using the WSAVA guidelines for histopathological evaluation of GI inflammation [1] and the simplified histopathologic scoring system for GI inflammation [33].

Four duodenal biopsy specimens were collected from thirty-seven (*n* = 7 HC/30 IBD) of these dogs using endoscopic biopsy forceps for the intestinal microbiota assessment. Both samples (duodenal specimens and rectal swabs) were collected in 2 mL sterile propylene cryotubes (Biosigma S.r.l., Cona, Italy), and were immediately frozen at −20 °C until further DNA extraction.

### 2.3. DNA Extraction

Genomic DNA was extracted from fecal samples and duodenal biopsies by mechanical and enzymatic lysis using a commercially available DNA extraction kit (DNeasy^®^Power Soil^®^ Pro–Kit, QIAGEN GmbH, Hilden, Germany), according to the manufacturer’s instructions [34,35]. In addition to the biopsies and fecal DNA samples, we extracted DNA from two negative controls (an unused sterile swab with nucleotide-free water in a sterile cryovial, and another from the culture media of the positive control) and a known concentration [1.7 × 10^9^ CFU/mL] of *Escherichia coli O 146* as a positive control. DNA concentrations from the extracts were determined using NanoDrop One (ThermoFisher Scientific, Waltham, MA, USA). DNA samples were stored at −20 °C until further analysis.

### 2.4. Library Preparation and Sequencing

DNA samples and controls were submitted to Sequencing and Bioinformatics Service (FISABIO, Valencia, Spain). An Illumina amplicon library was performed following the 16S rRNA gene Metagenomic Sequencing Library Preparation Illumina protocol (Cod. 15,044,223 Rev.A). The gene-specific primer sequences used in this protocol to amplify were selected from Klindworth et al. [36], targeting the 16S rRNA gene V3 and V4 hypervariable regions (341–F/805–R), and resulting in a single amplicon of approximately 460 pb. Overhang adapter sequences were used together with the primer pair sequencer for compatibility with Illumina index and sequencing adapters. Genomic DNA (5 ng/μL in 10 mM Tris pH 8.5) was used to initiate the protocol. After 16S rRNA gene amplification, the multiplexing step was performed using the Nextera XT Index Kit (FC–131–1096). We ran 1 μL of the PCR product on a Bioanalyzer DNA 1000 chip to verify the size. After size verification, the libraries were sequenced using a 2 × 300 pb paired-end run (MiSeq Reagent kit v3 [MS–102–3001]) on a MiSeq Sequencer according to the manufacturer’s instructions (Illumina, Inc., San Diego, CA, USA).

All raw sequences of this project have been deposited into the Sequence Read Archive (SRA) of the National Center for Biotechnology Information (NCBI) under accession number PRJNA905458.

### 2.5. Bioinformatic Analysis

Sequences were processed and analyzed using the Quantitative Insights Into Microbial Ecology software (QIIME) version 2.0. [37]. Denoising, paired-ends joining, and chimera depletion were performed starting from paired-ends data using the DADA2 pipeline generating amplicon sequence variants (ASVs) to ensure a sufficient depth to capture most features [38]. Taxonomy of ASVs were assigned using the Naïve Bayesian classifier integrated with QIIME2 plugins using the SILVA reference database (v.138.1) [39]. Bacterial taxa abundances were normalized to the total number of sequences in each sample and expressed as relative abundances. α-Diversity analysis (presented here as observed species and Shannon and inverse Simpson indexes) was utilized to estimate the samples’ diversity and richness using the R-package Vegan [40]. β-Diversity analyses were graphically explored by principal coordinates analysis (PCoA) using the “emperor” plugin of QIIME2 [41]. Permutational multivariate analysis of variance (PERMANOVA), applying 999 permutations, allowed us to establish which differences were statistically significant. Larger pseudo-F values indicate more pronounced group separation. For these analyses, the continuous variable age was categorized as young (<4), adult (4–8), or senior (>8), and the scores of the indexes were considered categories.

### 2.6. Statistical Analysis

Statistical analysis of signalment, clinical, endoscopic, and histopathological data of the dogs was performed using the commercially available statistical software SAS (version 9.4; SAS Inst., Cary, NC, USA). Differences were evaluated by T Student’s test in the case of a normal data distribution or by Wilcoxon’s signed rank test in the absence of a normal data distribution. The statistical analysis of the sequences obtained with Illumina MiSeq and the metadata file was analyzed using the RStatistics program [42] with the support of the FISABIO Bioinformatic Service. To determine differences in bacterial diversity indexes and relative proportions of bacterial taxa between groups, the Wilcoxon signed-rank test was used. The significance level was set at *p* < 0.05.

## 3. Results

### 3.1. Dogs

A total of forty-six dogs were included in the study. Twelve of these dogs were healthy, and thirty-four were diagnosed with IBD. Group compositions showed no difference in terms of age, sex, fertile status, breed, or body weight. However, IBD dogs showed a significantly lower body condition score (BCS; *p* = 0.001), and there were more healthy dogs living outdoors (33.33%) than dogs with IBD (5.88%) (*p* = 0.015). In addition, CIBDAI and CCECAI scores differed significantly between groups (*p* < 0.0001). The mean duration of the digestive process at the time of diagnosis was 27.5 ± 23.96 months (min: 5–max: 108; median: 20.5 months). A total of 61.76% (*n* = 21/34) of the IBD dogs had chronic or intermittent GI signs lasting more than a year. Data of all the dogs included in the study are summarized in Table 1 (more in detail in Supplementary Table S1).

### 3.2. Endoscopic and Histopathological Evaluation

When the WSAVA endoscopic index was applied, esophageal alterations were detected in 70.5% (*n* = 24/34), and gastric and duodenal lesions in 100% (*n* = 34/34) of the IBD dogs. When Slovak et al. indexes were applied, gastric lesions were observed in 94.11% (*n* = 32/34), and 100% in the duodenum of the IBD dogs. In both indexes, the scores assigned to these macroscopic lesions were statistically higher in the dogs with the disease than in the HC group. Regarding histopathological evaluation, all IBD dogs presented an infiltrate of lymphocytes and plasma cells in the duodenal lamina propria. This infiltrate was moderate in most cases (61.76%) and severe in the rest (38.24%) of the dogs. When applying both the WSAVA histopathological index and the abbreviate index proposed by Allenspach et al., only the duodenum showed statistically higher scores in the IBD group than in the HC dogs. The endoscopic and histopathological scores and comparative studies are shown in Table 2.

### 3.3. 16S-rRNA Sequencing

#### 3.3.1. Duodenal Biopsy Specimens

A total of 1,783,238 raw sequences were generated from the 37 duodenal specimens’ samples. The sequences median per sample was 48,196 (IQR [Interquartile range]: 30,224). After the filtering process, denoising, demultiplexing, and chimera removal, a total of 716,148 sequences were obtained (median: 19,355 per sample; IQR: 18,655). A total of 81.53% of the sequences were assigned to the bacterial domain and belonged to 17 phyla, 18 classes, 71 orders, 123 families, 248 genera, and 351 bacterial species. Table 3 summarizes the relative proportions of duodenal mucosa-associated biopsies.

Regarding the phylum Actinobacteria, a decrease in the class Rubrobacteria, order Rubrobacterales, family Rubrobacteriaceae, and genus *Rubrobacter* (*p* = 0.002) was observed in the IBD dogs. Furthermore, a reduction of Cyanobacteria was observed in the IBD dogs (*p* = 0.010). Within this phylum, a decrease in the class Vampirivibrionia, order Obscuribacterales, and family Obscuribacteraceae was observed (*p* = 0.005). Finally, the IBD dogs showed a reduction in the relative abundance of the family Neisseriaceae (*p* = 0.04) and genus *Conchiformibius* (*p* = 0.003) belonging to the class Gammaproteobacteria.

**Figure 1:**
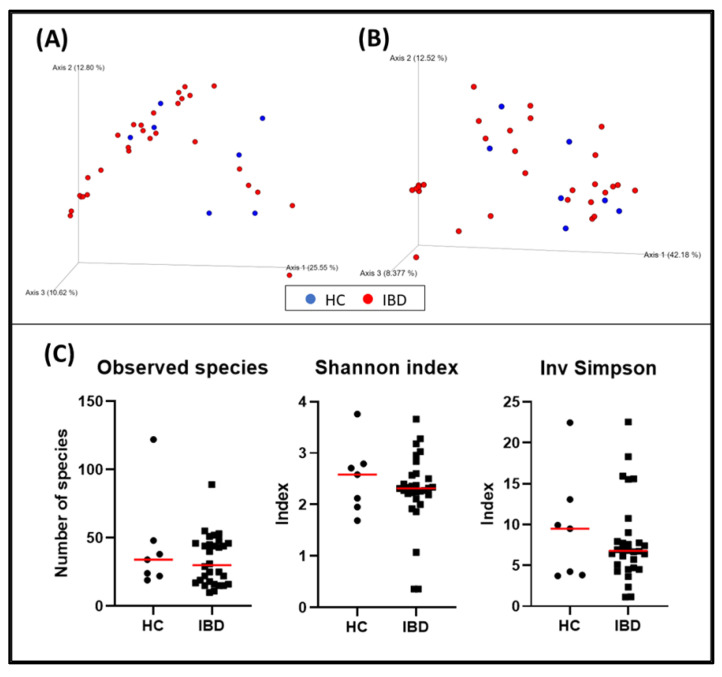
Bacterial diversity measures of duodenal samples: The dimensional representation of principal coordinate analysis (PCoA) plots of (**A**) unweighted and (**B**) weighted UniFrac distances of 16S rRNA genes. Duodenal biopsy specimens from dogs with IBD were not separated from those obtained from healthy dogs (PERMANOVA, *p* = 0.358; pseudo–F: 1.07). (**C**) Comparisons of α–diversity: observed species (*p* = 0.600), Shannon index (*p* = 0.684), and inverse Simpson index (*p* = 0.742). Red lines represent the median for each measure. HC, healthy control dogs; IBD, inflammatory bowel disease dogs.

When evaluating α-diversity, there were no statistically significant differences regarding the detected bacterial species (HC = median: 34 [19–122]; IBD = median: 30 [10–89]; *p* = 0.600) or the Shannon (HC = median: 2.58 [1.69–3.76]; IBD = median: 2.32 [0.36–3.66]; *p* = 0.684) and inverse Simpson (HC = median: 9.49 [3.71–22.47], IBD = median: 6.78 [1.13–22.56]; *p* = 0.742) indexes of the duodenal biopsies (Figure 1).

The bacterial composition of the duodenal samples from dogs with IBD was not significantly different from the one observed in healthy dogs (*p* = 0.358; pseudo–F: 1.07) (Figure 1, and Table 4). The rest of the variables collected were also evaluated, and fertile status (*p* = 0.034, pseudo–F: 3.22), weight (*p* = 0.014, pseudo–F: 2.58), and histopathological indexes including WSAVA and the abbreviated showed clustering (Table 4).

#### 3.3.2. Fecal Microbiota Communities

A total of 7,447,439 raw sequences were generated from the 46 fecal samples. The sequences median per sample was 145,245 (IQR: 51,323). After the filtering process, denoising, demultiplexing, and removal of chimeric sequences, a total of 4,878,308 sequences were obtained (median: 96,777 per sample; IQR:32,584). A total of 99.95% of these sequences were assigned to the bacterial domain and included 13 phyla, 18 classes, 47 orders, 83 families, 218 genera, and 374 bacterial species. The most abundant phyla in the fecal samples in our study were Firmicutes (68.68%), Bacteroidetes (10%), Fusobacteria (6.89%), Actinobacteria (5.66%), Campylobacterota (4.75%), and Proteobacteria (3.97%). None of the remaining phyla had a relative abundance higher than 0.01%. Table 5 summarizes the relative proportions of fecal bacterial taxa differencing between groups.

At the phylum and class levels, there were no significant differences between the IBD and healthy dogs. However, significant differences were identified at lower phylogenetic levels. Within the phylum Bacteroidetes, a reduced proportion of some taxa including family Prevotellaceae (*p* = 0.005), genera *Prevotella* (*p* = 0.002), and *Prevotellaceae Ga6A1 group* (*p* = 0.006) was observed in the IBD dogs.

Firmicutes was the most abundant phylum among groups. At lower levels, a reduction in the relative abundance of order Erysipelotrichales (*p* = 0.019) was observed, specifically, in the relative abundance of the family Erysipelotrichaceae (*p* = 0.005), and the genera *Allobaculum* (*p* = 0.003). Within the family Erysipelatoclostridiaceae, a reduction in the genus *Candidatus* Stoquefichus was detected (*p* < 0.001). Furthermore, some taxa belonging to class Clostridia were reduced: the genera *Lachnospiraceae NK4A136 group* (*p* = 0.015) and *Sellimonas* (*p* = 0.042), Oscillospirales (*p* = 0.037), *UCG–005* (*p* < 0.001), and other genera belonging to the Ruminococcaceae family, *Faecalibacterium* (*p* = 0.028), and *Fournierella* (*p* = 0.034). Moreover, a reduction in the relative abundance of the order Acidaminococcales, family Acidaminococcaceae, and genus *Phascolarctobacterium* (*p* = 0.001) was found in the fecal samples of the IBD dogs. On the other hand, an increased relative abundance of Enterococcaceae (*Enterococcus*; *p* = 0.003) and *Streptococcaceae* (*Streptococcus*; *p* = 0.021) was observed in the IBD dogs.

Regarding Proteobacteria, there was a reduction in the relative proportion of order Aeromonadales, family Succinivibrionaceae (*p* = 0.026), and the genus *Succinivibrio* (*p* = 0.031). On the contrary, the IBD dogs showed an increase in the relative proportion of Enterobacterales (*p* = 0.027), Enterobacteriaceae (*p* = 0.008), and *Escherichia–Shigella* (*p* = 0.011).

Finally, no differences were observed in lower levels in the phyla Campylobacterota or Fusobacteria. However, a reduction close to statistical significance was found in Fusobacteria (*p* = 0.052) in IBD dogs.

**Figure 2:**
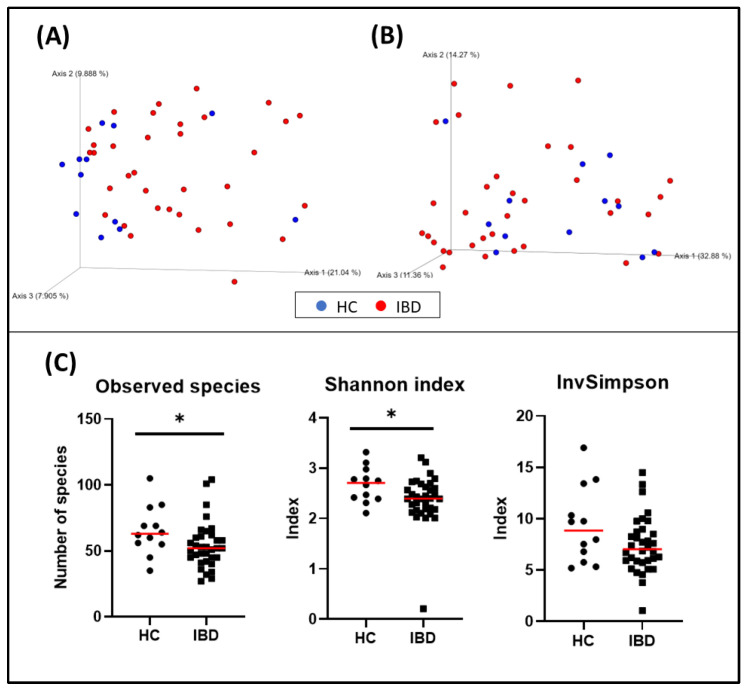
Bacterial diversity measures of fecal samples: The dimensional representation of principal coordinate analysis (PCoA) plots of (**A**) unweighted and (**B**) weighted UniFrac distances of 16S rRNA genes. Fecal samples from dogs with IBD were separated from those obtained from healthy dogs (PERMANOVA, *p* = 0.006; pseudo–F: 4.83). (**C**) Comparisons of α-diversity: observed species (*p* = 0.031), Shannon index (*p* = 0.039), and inverse Simpson index (*p* = 0.140). Red lines represent the median for each measure. HC, healthy control dogs; IBD, inflammatory bowel disease dogs; * *p* < 0.05.

When evaluating α-diversity, a significant difference in the number of bacterial species (HC = median: 63 [35–105], IBD = median: 52 [27–104]; *p* = 0.031), and the Shannon index (HC = median: 2.71 [2.11–3.32], IBD = median: 2.39 [0.21–3.21]; *p* = 0.039) was observed. The inverse Simpson was also higher in the HC group than in the IBD group, but it was not statistically significant (HC = median: 8.84 [5.19–16.91], IBD = median: 7.03 [1.06–14.49]; *p* = 0.140) (Figure 2).

The bacterial composition of the fecal microbiota from dogs with IBD was significantly different from that observed in healthy dogs (*p* = 0.006, pseudo–F: 4.83) (Figure 2, and Table 6). Other variables were also evaluated and weight, living with other pets, the time elapsed from the onset of the disease and the sampling, and the CIBDAI index also showed clustering (Table 6).

#### 3.3.3. Duodenal Biopsies vs. Fecal Samples

**Figure 3:**
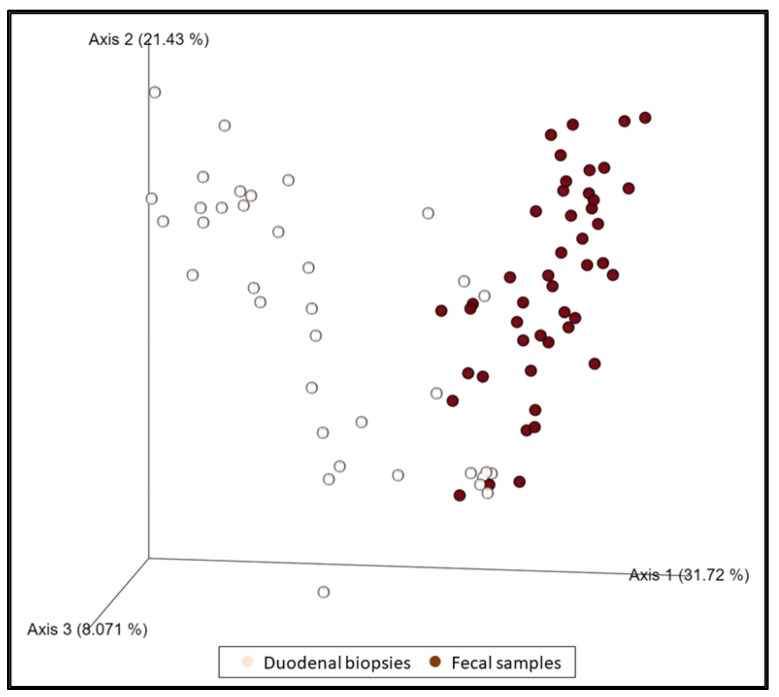
The dimensional representation of principal coordinate analysis (PCoA) plots of weighted UniFrac distances of 16S rRNA genes. Fecal samples were separated from duodenal samples (PERMANOVA, *p* = 0.001, pseudo–F: 17.39).

When all duodenal specimens (*n* = 37) and fecal samples (*n* = 46) were compared regardless of the group of dogs, they were shown to be statistically different (*p* = 0.001, pseudo–F: 17.39) (Figure 3). Some changes in the relative abundance were observed in both biopsies and feces (decrease in the genus *Eubacterium nodatum group*, and *Prevotella*) in a statistically significant manner. Likewise, an increase in *Escherichia–Shigella*, *Enterococcus*, and *Streptococcus* was observed in biopsies which, although not statistically significant, is reflected in a significant increase in fecal samples.

## 4. Discussion

To our knowledge, this is the first study that assesses the bacterial composition and diversity in both duodenal biopsy specimens and rectal fecal samples in dogs with IBD. We provide valuable information regarding signalment, environmental factors, the time elapsed from the onset of clinical signs and the collection of samples, as well as clinical activity indexes, and endoscopic and histologic findings of the GI tract of these dogs.

Most bacterial sequences identified in the canine GI tract belong to the phyla Firmicutes, Fusobacteria, Bacteroidetes, Proteobacteria, and Actinobacteria [16,43], and, as expected, were also identified in the samples of this study. Firmicutes (68.68%) was the most abundant phylum in the fecal samples, while Proteobacteria (43.68%) and Firmicutes (37.37%) were the most abundant phyla in the duodenal biopsies. These findings are consistent with others previously reported, where Proteobacteria was the most abundant phylum in the duodenal biopsies [44] and Firmicutes the most abundant in canine fecal samples [22,45].

Previous studies characterizing the canine gut microbiota from duodenal biopsies showed shifts in microbial communities and its reduction of diversity in dogs with IBD [34,44,46]. Specifically, the phylum Proteobacteria is the most consistently associated with IBD, as well as the reduction in some taxa belonging to Clostridiales, also frequently reported [44,46]. However, in our study, only a few taxa were significantly lower in the mucosa samples of IBD dogs in comparison with healthy dogs. The dogs of the IBD group presented a lower relative abundance of the class Rubrobacteria, order Rubrobacterales, family Rubrobacteriaceae, and genus *Rubrobacter* belonging to the Actinobacteria phylum. To the authors’ knowledge, this is the first time that *Rubrobacter* has been described in this type of sample in the canine species but has been already detected in the stomach of rats [47], in the ileum of chicken broilers [48], and in human fecal samples [49]. We also observed a decrease in the relative abundance of the Cyanobacteria phylum, including a reduction in the class Vampirivibrionia, order Obscuribacterales, and the family Obscuribacteraceae. Finally, a decrease in the family Neisseriaceae and, specifically, in *Conchiformibius*, was detected in the duodenal biopsies of the dogs with IBD. This finding is contrary to what was previously observed in duodenal biopsies, where this genus (*Conchiformibius*), belonging to the phylum Proteobacteria, was only detected in biopsies from dogs with IBD [46].

It is important to highlight that the differences between our study and those described above could be due to methodological factors (i.e., different targeted regions of the 16S rRNA gene, type of sequencing, extraction method, and continuous updating of the reference database), which makes a comparison between studies difficult [21]. In this sense, one potential limitation of our study is the small number of dogs included in the control group, even though our study comprises the largest duodenal biopsy sample size of dogs with IBD and presented a similar HC group of dogs compared with previous studies [44,46]. In addition, it should also be considered that small intestinal microbiota can contribute to clinical signs even if it is normal in composition when there is an abnormal or increased content in the intestinal lumen [21]. In dogs with IBD, the inflammation leads to the malabsorption of nutrients, and consequently, to the abnormal bacterial conversion of luminal substances by the normal microbiota. Thus, a complementary approach (i.e., taxonomic, and functional) that could explain this lack of alterations in the mucosal-associated microbiota of the duodenum is highly recommended.

When assessing the β-diversity of the duodenal samples, the microbiota showed no clustering due to the disease, but, interestingly, it was observed when the histopathological indexes were applied. In addition, clustering was found when the microbiota was analyzed based on weight and fertile status. Gonadectomy may have an impact on the microbiota because sexual hormones have been associated with certain bacteria in the gut and this could lead to different compositions between sex, as described by some authors [50,51]. However, no clustering was observed in the microbiota regarding sex, and this finding was neither observed in the fecal samples. Further investigations with a bigger sample size are needed to determine whether the sexual hormones could affect the composition of the microbiota in the dog species at this level, as previous studies fail to demonstrate any association in fecal samples [50]. Moreover, some authors have described that the size of the animal can influence the composition of the microbiota [52].

Although many studies have focused on the evaluation of naturally voided feces, we collected samples from the rectum before the endoscopy in all the dogs to avoid potential contaminations or different periods or conditions of storage from the collection of the samples by the owner. Previous studies have shown that compositional changes in dogs with IBD consisted of a reduction in the relative abundances of the Bacteroidetes and Firmicutes phyla and *Fusobacterium* spp. and an increased abundance of Proteobacteria and Actinobacteria when compared with healthy dogs [19,22,23,24,25]. Despite our study showing similar trends (higher proportions of Proteobacteria and Actinobacteria and lower for Bacteroides and Fusobacteria), no difference was observed at the phyla level in our study when comparing healthy dogs and dogs with IBD. This discrepancy was also described by Omori and coworkers when comparing fecal samples from healthy and IBD-affected dogs [45].

We observed a reduction in taxa within the phylum Bacteroidetes, including the family Prevotellaceae and genera *Prevotella* and the *Prevotellaceae Ga6A1 group*. These bacteria are producers of short-chain fatty-acids (SCFAs) such as acetate, butyrate, and propionate; they have beneficial effects on the host, and have been previously linked to non-IBD dogs [19,23]. Firmicutes was the most abundant phylum in the fecal samples, and some shifts in the taxa it comprises were observed. A reduction in the relative abundance of Erysipelotrichales order, including the Erysipelotrichaceae family and the genus *Allobaculum*, as well as the *Candidatus* Stoquefichus genus (Erysipelatoclostridiaceae family), was also observed. Again, these taxa were associated previously with dogs non-affected by IBD and are usually considered to be commensal bacteria [19,23,53,54].

Another important bacterial taxon within the Firmicutes phylum and, in this case, Clostridia class and Lachnospirales order, is Lachnospiraceae [19,54]. This family was the most abundant detected in the fecal samples. In previous studies conducted in dogs with IBD, a decrease in members of this family, as well as in the Ruminococcaceae family, was observed [23,24]. Similar to these findings, we observed a decrease in the *Lachnospiraceae NK4A136 group* and *Sellimonas*, and other genera belonging to the Ruminococcaceae family, *Faecalibacterium*, and *Fournierella*. The genus *Faecalibacterium* is known for its anti-inflammatory properties in the human species and has been considered a potential biomarker of improved GI functionality for dogs, showing a decrease in IBD [19,22,55,56,57]. Ruminococcaceae and Lachnospiraceae are anaerobic bacterial families that are well known due to their role as central SCFA-producing bacteria. Thus, the simultaneous decline of all these taxa causes a reduction in the availability of SCFAs, which constitute the main energy source for colonocytes [24]. Moreover, a reduction in Acidaminococcaceae, represented by the *Phascolarctobacterium* genus, was observed in the dogs with IBD in this study. This genus has been shown to be related to the synthesis of propionate, which again could be a key factor involved in intestinal homeostasis [58]. Within the Lactobacillales order, a marked increase was observed in the Enterococcaceae family and *Enterococcus* genus, as well as in the Streptococcaceae family and *Streptococcus* genus. These genera are heterofermentative bacteria that can produce lactic acid and have been also previously associated with dogs affected by IBD [19,22,23,59]. Furthermore, *Streptococcus* overgrowths in maldigestion [60] have been considered a hallmark of canine dysbiosis [19,55,61].

Within the Proteobacteria phylum, a decrease was observed in the Aeromonadales order, specifically in the Succinivibrionaceae family and *Succinivibrio* genus in the dogs with IBD of this study. *Succinivibrio* has been described in high abundance in the gut of high-starch-fed cattle, where it plays a role in propionate production through the production of upstream succinate [62]. However, there are scarce studies detecting *Succinivibrio* in canine fecal samples and their functions remain unknown [63]. The Enterobacterales order, Enterobacteriaceae family, and genus *Escherichia–Shigella* were also increased in the dogs with IBD. This finding was previously observed in fecal samples of dogs with this enteropathy [22,23]. In fact, *Escherichia coli* has been considered a hallmark of dysbiosis due to its pro-inflammatory properties not only in dogs [55,56], but also in cats [64] and humans [19].

When evaluating α-diversity, the dogs with IBD showed lower diversity in terms of richness and abundance of species. These findings were previously described in fecal samples of IBD [19,24], and have been considered a biomarker of gastrointestinal dysfunctionality [56]. Within the β-diversity, clustering was observed by the condition of health or disease, i.e., fecal microbiota in dogs with IBD was significantly different from the one detected in healthy dogs. Additionally, we also found differences regarding weight, living with other pets, the period from the onset to the diagnosis, and the CIBDAI score. The clustering found in the CIBDAI score could be related to the health condition, because all the animals (healthy dogs and dogs with IBD) were included in the analysis. On the other hand, the weight was not only clustered in duodenal microbiota, but also in fecal microbiota. There was no difference regarding body weight between groups. However, there was a significant decrease in the BCS in the IBD group, which could partially explain this difference. Finally, most of the healthy dogs lived with other pets in the household, while less than 50% of the IBD group did not. Thus, the clustering observed when assessing β-diversity could be mainly explained because of the health condition.

Most sequencing-based taxonomic studies have been focused on the fecal microbiota because this is the most accessible type of sample in veterinary clinical practice [21]. Unlike in the human species, canine fecal samples present most of the intestinal mucosa-associated bacterial taxa [19]. In fact, our study clearly demonstrates that most of the higher taxa present in biopsies are also present in fecal samples. However, fecal samples do not provide complete information regarding the composition of the small intestine microbiota in terms of the potential presence of mucosa-adherent or entero-invasive bacteria [21].

One of the main findings of our study was that while differences in composition and diversity were found in fecal samples when comparing healthy dogs and dogs with IBD, only a few changes in the relative abundance of some taxa were observed in the duodenal-associated microbiota using the same approach. This finding is, at least partially, reasonable, because they constitute a different type of sample, and it is known that bacteria vary along the canine gastrointestinal tract [43]. However, the localization of the inflammatory process would have made expectable a higher degree of changes in bacterial microbiota at this level.

When biopsy samples and fecal samples were compared regardless of the condition, they were unrelated. However, it should be noted that some changes described in this study are observed in both biopsies and feces. Thus, a decrease in the genera *Eubacterium nodatum group* and *Prevotella* were detected in a statistically significant manner. Nonetheless, only the *Prevotella* genus is represented in at least 50% of the canine fecal samples. Likewise, an increase in *Escherichia–Shigella*, *Enterococcus*, and *Streptococcus* is observed in biopsies which, although not statistically significant, is reflected in a significant increase in fecal samples. This makes these genera an important target as a biomarker of the disease.

Finally, some factors that should be considered when analyzing the results of this study are the diet [65], and the washout time of previous treatments [66,67]. Several studies have shown the effect of dietary interventions on canine GI microbiota. The ingested food serves as a substrate for the intestinal microbiota of the host and plays an important role in defining its composition and metabolism. In our study, healthy dogs were fed maintenance commercial diets, while dogs with IBD were fed a hydrolyzed protein-based diet. A hydrolyzed protein-based diet, formulated to reduce immunogenicity and with highly digestible ingredients, does not seem to significantly affect the gut microbiota of healthy dogs. However, they have shown to partially recover the altered microbiota in chronic enteropathies [34,68]. Thus, these differences in the diet between groups constitute an important limitation in our study in the comparison of the intestinal microbiota. However, the fact that they were privately owned makes it difficult to assess the effect of the pre-study diets in most cases, but, at the same time, results are more extrapolated to the dog population [69].

On the other hand, due to the nature of our center (Referral Hospital), most of the cases had been previously attended in other private clinics, and different protocols and treatments had been applied before. For this study, we adopted a 4-week washout based on previous studies. However, if antibiotics such as metronidazole have been used, some dogs could still present some alterations in bacterial composition [66]. Therefore, at least some of the differences in microbiota compositions among dogs with IBD could be explained by the different previously applied treatments.

## 5. Conclusions

Our study shows that the composition and diversity of fecal microbiota in dogs with IBD were significantly different from healthy dogs. However, only a few bacterial taxa shifts were observed when assessing duodenal-associated microbiota through analysis of duodenal biopsies specimens. Thus, even if the bowel inflammation mainly affects the small intestine in the IBD-affected dogs of the study, fecal specimens constitute a better sample, due not only to their easy availability, but also in terms of searching for bacterial taxa as biomarkers for canine IBD. Future studies encompassing multi-omics approaches should evaluate the relationship between bacterial composition and diversity and the metabolome to unravel the relationship between the microbiota and the pathophysiology of IBD in canine species to assess and modulate the microbiome in the disease.

## Figures and Tables

**Table 1:** Comparison of signalment, epidemiological data, and clinical scores of the dogs enrolled in the study.

Variables	HC (*n* = 12)	IBD (*n* = 34)	*p*-Value
Age (years; mean ± SD)	5.31 ± 3.09	6.05 ± 3.47	0.519
Sex (male/female)	7/5	15/19	0.487
Fertile status (spayed or neutered/entire)	8/4	21/13	1.000
Breed (pure/mixed)	7/5	24/10	0.436
Weight (kg); median [range])	13.85 [4.50–32.80]	11.80 [2.30–44]	0.763
BCS (1–9); median [range])	5.50 [5–7]	4.00 [2–7]	0.001 *
Living with other pets (yes/no)	7/5	10/24	0.093
Habitat (indoor/50–50/outdoor)	8/0/4	25/7/2	0.025 *
CIBDAI (median [range])	0 [0]	6.5 [3–10]	<0.0001 *
CCECAI (median [range])	0 [0]	7 [3–12]	<0.0001 *
Duodenal biopsies/fecal samples	7/12	30/34	na

* *p*-value was significant when < 0.05; BCS, body condition score; CIBDAI, canine IBD activity index; CCECAI, canine chronic enteropathy clinical activity index; IBD, inflammatory bowel disease; duodenal biopsies and fecal samples refer to the total number of dogs from which the samples were retrieved for microbiota analysis; na, not applied.

**Table 2:** Comparison of macroscopic and histological findings using different scores in the dogs enrolled in the study.

Evaluation		Mean ± SD		
**Macroscopic**	**∑ Values (Range)**	**HC (*n* = 12)**	**IBD (*n* = 34)**	***p*–Value**
WSAVA [1]	Esophagus (0–27)	0.08 ± 0.29	1.62 ± 1.60	<0.0001 *
	Stomach (0–33)	2.70 ± 1.83	5.44 ± 2.18	0.001 *
	Duodenum (0–33)	4.67 ± 2.84	8.32 ± 2.69	0.0002 *
Slovak et al. [32]	Quantitative stomach (0–6)	0.5 ± 0.53	1.79 ± 1.01	<0.0001 *
	Quantitative duodenum (0–8)	2.08 ± 1.08	3.44 ±1.31	0.002 *
	Qualitative stomach (0–3)	0.5 ± 0.53	1.59 ± 0.74	<0.0001 *
	Qualitative duodenum (0–4)	1.67 ± 0.78	2.38 ± 0.82	0.001 *
**Histopathologic**	**∑ Values (Range)**	**HC (*n* = 12)**	**IBD (*n* = 34)**	***p*-Value**
WSAVA [1]	Stomach (0–27)	3.71 ± 2.29	4.59 ± 11.88	0.301
	Duodenum (0–27)	4.73 ± 2.45	11.88 ± 3.76	<0.0001 *
Allenspach et al. [33]	Stomach (0–15)	2.86 ± 1.21	3.29 ± 1.34	0.430
	Duodenum (0–18)	3.91 ± 1.92	9.18 ± 2.83	<0.0001 *

* *p*-value was significant when < 0.05; WSAVA, World Small Animal Veterinary Association.

**Table 3:** Relative proportions of predominant bacterial taxa in the duodenal samples.

Relative Abundance % (Min–Max) of Sequences			
Phylum/Class/Order/Family/*Genus*	HC (*n* = 7)	IBD (*n* = 30)	*p*-Value
Actinobacteria	4.86 (0.24–13.85)	8.23 (0.00–19.16)	0.362
Actinobacteria	4.60 (0.23–13.85)	3.84 (0.00–17.87)	0.362
Actinomycetales	0.57 (0.00–3.19)	0.46 (0.00–5.90)	0.518
Actinomycetaceae	0.57 (0.00–3.19)	0.46 (0.00–5.90)	0.518
*Actinomyces*	0.57 (0.00–3.19)	0.46 (0.00–5.90)	0.518
Bifidobacteriales	2.78 (0.00–13.85)	2.28 (0.00–12.10)	1.000
Bifidobacteriaceae	2.78 (0.00–13.85)	2.28 (0.00–12.10)	1.000
*Bifidobacterium*	2.78 (0.00–13.85)	2.28 (0.00–12.1)	1.000
Corynebacteriales	1.09 (0.00–6.48)	0.42 (0.00–3.06)	0.746
Rubrobacteria	0.18 (0.00–1.06)	0.03 (0.00–0.64)	0.002 *
Rubrobacterales	0.18 (0.00–1.06)	0.03 (0.00–0.64)	0.002 *
*Rubrobacteriaceae*	0.18 (0.00–1.06)	0.03 (0.00–0.64)	0.002 *
*Rubrobacter*	0.18 (0.00–1.06)	0.03 (0.00–0.64)	0.002 *
Bacteroidetes	6.83 (0.00–20.52)	6.81 (0.00–34.39)	0.954
Bacteroidia	6.74 (0.00–20.52)	6.80 (0.00–34.39)	1.000
Bacteroidales	1.05 (0.00–3.72)	2.85 (0.00–34.16)	0.331
Flavobacteriales	5.67 (0.00–20.52)	3.79 (0.00–33.84)	0.493
Weeksellaceae	5.46 (0.00–19.83)	3.66 (0.00–3.67)	0.419
*Chryseobacterium*	2.30 (0.00–11.29)	3.44 (0.00–33.67)	0.884
Cyanobacteria	1.86 (0.00–6.48)	0.44 (0.00–5.73)	0.010 *
Vampirivibrionia	0.58 (0.00–1.82)	0.04 (0.00–0.83)	0.005 *
Obscuribacterales	0.58 (0.00–1.82)	0.04 (0.00–0.83)	0.005 *
Obscuribacteraceae	0.58 (0.00–1.82)	0.04 (0.00–0.83)	0.005 *
Firmicutes	26.89 (0.38–60.64)	39.82 (1.02–99.71)	0.323
Bacilli	16.57 (0.11–60.19)	21.06 (0.08–57.44)	0.362
Bacillales	4.47 (0.00–14.81)	5.75 (0.00–27.36)	0.786
Bacillaceae	4.47 (0.00–14.81)	5.75 (0.00–27.36)	0.786
*Anaerobacillus*	2.82 (0.00–10.41)	2.41 (0.00–5.90)	0.953
*Bacillus*	1.65 (0.00–6.48)	3.28 (0.00.18.24)	0.389
Lactobacillales	8.39 (0.03–30.13)	11.97 (0.00–40.64)	0.522
Lactobacillaceae	4.85 (0.00–30.13)	3.15 (0.00–24.66)	0.905
*Lactobacillus*	4.85 (0.00–30.13)	3.15 (0.00–24.66)	0.905
Streptococcaceae	2.14 (0.00–7.14)	6.73 (0.00–23.12)	0.340
*Streptococcus*	2.14 (0.00–7.14)	6.73 (0.00–23.12)	0.340
Staphylococcales	3.64 (0.00–15.26)	0.785 (0.00–4.29)	0.202
Gemellaceae	0.54 (0.00–2.38)	0.28 (0.00–2.52)	0.275
*Gemella*	0.54 (0.00–2.38)	0.28 (0.00–2.52)	0.275
Staphylococcaceae	3.11 (0.00–15.26)	0.50 (0.00–4.29)	0.134
*Staphylococcus*	3.10 (0.00–15.26)	0.50 (0.00–4.29)	0.256
Clostridia	9.87 (0.10–26.19)	18.64 (0.00–99.63)	0.627
Clostridiales	3.35 (0.00–21.03)	3.49 (0.00–56.33)	0.767
Clostridiaceae	3.35 (0.00–21.03)	3.49 (0.00–56.33)	0.767
*Clostridium sensu stricto 1*	0.39 (0.00–1.50)	3.33 (0.00–52.55)	0.899
Lachnospirales	1.42 (0.00–4.63)	9.28 (0.00–32.81)	0.284
Lachnospiraceae	1.37 (0.00–4.63)	9.24 (0.00–32.81)	0.319
Peptostreptococcales–Tissierellales	3.67 (0.00–14.02)	5.53 (0.00–92.26)	0.876
Peptostreptococcaceae	2.95 (0.00–14.02)	5.44 (0.00–92.26)	0.740
*Romboutsia*	2.25 (0.00–14.02)	3.37 (0.00–92.26)	0.407
Proteobacteria	57.14 (15.83–77.81)	40.54 (0.27–6.65)	0.222
Alphaproteobacteria	7.61 (0.58–23.33)	6.46 (0.00–30.02)	0.574
Caulobacterales	1.92 (0.00–9.93)	0.59 (0.00–3.80)	0.441
Caulobacteraceae	1.92 (0.00–9.93)	0.59 (0.00–3.80)	0.441
*Phenylobacterium*	1.92 (0.00–9.93)	0.59 (0.00–3.73)	0.441
Rhizobiales	2.45 (0.07–8.12)	2.22 (0.00–11.47)	0.402
Beijerinckiaceae	1.03 (0.00–5.00)	0.72 (0.00–5.54)	0.885
*Methylobacterium–Methylorubrum*	0.72 (0.00–4.78)	0.57 (0.00–5.54)	0.327
Rhizobiaceae	0.76 (0.00–2.56)	0.92 (0.00–6.56)	0.885
Xanthobacteraceae	0.60 (0.00–1.14)	0.54 (0.00–4.66)	0.163
*Bradyrhizobium*	0.41 (0.00–1.02)	0.25 (0.00–3.99)	0.059
Sphingomonadales	2.20 (0.37–6.35)	3.22 (0.00–17.17)	0.472
Sphingomonadaceae	2.20 (0.37–6.35)	3.22 (0.00–5.29)	0.472
*Sphingomonas*	2.00 (0.00–6.35)	1.37 (0.00–14.44)	0.132
Gammaproteobacteria	49.53 (13.41–73.79)	34.08 (0.27–86.29)	0.135
Burkholderiales	6.00 (1.46–12.16)	4.25 (0.00–18.54)	0.180
Alcaligenaceae	0.62 (0.00–1.90)	0.85 (0.00–4.73)	0.950
Burkholderiaceae	0.79 (0.00–2.48)	0.98 (0.00–10.67)	0.639
Comamonadaceae	1.99 (0.00–9.76)	1.01 (0.00–4.08)	0.352
*Delftia*	1.05 (0.00–5.30)	0.74 (0.00–4.08)	0.485
Neisseriaceae	1.68 (0.03–8.20)	0.63 (0.00–5.32)	0.004 *
*Conchiformibius*	1.30 (0.00–5.80)	0.41 (0.00–5.10)	0.003 *
Enterobacterales	2.78 (0.00–18.12)	3.35 (0.00–15.84)	0.534
Enterobacteriaceae	2.78 (0.00–18.12)	3.30 (0.00–15.84)	0.534
*Escherichia–Shigella*	1.92 (0.00–12.43)	2.68 (0.00–15.84)	0.471
Pasteurellales	3.26 (0.00–21.77)	1.65 (0.00–18.07)	0.383
Pasteurellaceae	3.26 (0.00–21.77)	1.65 (0.00–18.07)	0.383
Pseudomonadales	28.70 (3.17–56.64)	14.33 (0.00–51.53)	0.071
Pseudomonadaceae	26.38 (0.00–56.64)	12.85 (0.00–51.53)	0.174
*Pseudomonas*	26.38 (0.00–56.64)	12.85 (0.00–51.53)	0.167
Xanthomonadales	8.49 (0.00–24.44)	10.16 (0.00–37.02)	1.000
Xanthomonadaceae	8.49 (0.00–24.44)	10.16 (0.00–37.02)	1.000
*Stenotrophomonas*	8.36 (0.00–24.44)	9.94 (0.00–36.91)	0.968

IBD, inflammatory bowel disease; taxa present in at least 50% of the dogs (in any group) were included in this table. * *p* value < 0.05 using the Wilcoxon signed-rank test.

**Table 4:** Permutational multivariate analysis of variance (PERMANOVA) in bacterial compositions of duodenal biopsy specimens considering different variables.

Variables		Grouping	Pseudo–F	*p*-Value
Clinical condition		HC vs. IBD	1.07	0.358
Age		Young (<4)/adult (4–8)/senior (>8)	1.18	0.346
Sex		Male/female	0.60	0.650
Fertile status		Spayed or neutered/entire	3.22	0.034 *
Breed		Pure-breed/mixed-breed	1.03	0.393
Weight		Small (<10 Kg)/medium-size (10–20 Kg)/large-size (>20 Kg)	2.58	0.014 *
BCS		Low (1–4)/normal (5)/high (6–9)	0.57	0.750
Living with other pets		Yes/no	0.58	0.663
Habitat		Indoor/50–50/outdoor	0.64	0.680
Clinical onset–diagnosis		NA/more than a year/less than a year	1.24	0.317
Clinical activity indexes				
CIBDAI [31]		NA, clinically insignificant, mild, moderate, severe	0.72	0.679
CCECAI [28]		NA, clinically insignificant, mild, moderate, severe	0.67	0.769
Endoscopic indexes				
Slovak et al. [32]	Stom. Quan.	Scores (0–4)	1.01	0.455
	Stom. Qual.	Scores (0–3)	1.26	0.284
	Duod. Quan.	Scores (0–6)	0.45	0.946
	Duod. Qual.	Scores (0–4)	0.60	0.759
WSAVA [1]	Esophagus	Scores (0–6)	1.13	0.354
	Stomach	Scores (0–12)	0.98	0.500
	Duodenum	Scores (0–15)	0.66	0.864
Histopathological indexes				
WSAVA [1]	Stomach	Scores (0–8)	2.49	0.007 *
	Duodenum	Scores (0–18)	1.84	0.046 *
Abbreviated [33]	Stomach	Scores (0–6)	2.08	0.037 *
	Duodenum	Scores (0–14)	1.73	0.035 *

* *p*–value was significant when < 0.05; BCS, body condition score; CIBDAI, canine IBD activity index; CCECAI, canine chronic enteropathy clinical activity index; duod., duodenum; HC, healthy control; IBD, inflammatory bowel disease; NA, not applied; qual., qualitative; quant., quantitative; stom., stomach; WSAVA, World Small Animal Veterinary Association.

**Table 5:** Relative proportions of predominant bacterial taxa in the fecal samples.

Relative Abundance % (Min–Max) of Sequences			
Phylum/Class/Order/Family/*Genus*	HC (*n* = 12)	IBD (*n* = 34)	*p*-Value
Actinobacteria	3.87 (0.02–10.25)	6.29 (0.26–25.33)	0.216
Actinobacteria	0.89 (0.00–4.89)	0.90 (0.00–9.42)	0.910
Actinomycetales	0.02 (0.00–0.10)	0.27 (0.00–4.04)	0.105
Actinomycetaceae	0.02 (0.00–0.10)	0.27 (0.00–4.04)	0.105
*Actinomyces*	0.01 (0.00–0.07)	0.13 (0.00–1.38)	0.058
Corynebacteriales	0.11 (0.00–0.56)	0.47 (0.00–8.11)	0.150
Corynebacteriaceae	0.11 (0.00–0.56)	0.47 (0.00–8.11)	0.150
*Corynebacterium*	0.11 (0.00–0.56)	0.47 (0.00–8.11)	0.150
Coriobacteriia	2.97 (0.00–7.21)	5.39 (0.06–25.17)	0.165
Coriobacteriales	2.97 (0.00–7.21)	5.39 (0.06–25.17)	0.165
Coriobacteriaceae	2.78 (0.00–7.21)	5.19 (0.06–25.17)	0.173
*Collinsella*	2.78 (0.00–7.21)	5.19 (0.06–25.17)	0.173
Eggerthellaceae	0.19 (0.00–0.76)	0.18 (0.00–0.70)	0.380
*Slackia*	0.18 (0.00–0.68)	0.15 (0.00–0.70)	0.262
Bacteroidetes	13.72 (0.05–38.83)	8.69 (0.00–26.66)	0.150
Bacteroidia	13.72 (0.05–38.83)	8.69 (0.00–26.66)	0.150
Bacteroidales	13.72 (0.05–38.83)	8.69 (0.00–26.66)	0.150
Bacteroidaceae	4.80 (0.02–18.53)	4.66 (0.00–26.66)	0.608
*Bacteroides*	4.80 (0.02–18.53)	4.66 (0.00–26.66)	0.608
Prevotellaceae	8.76 (0.01–32.38)	3.59 (0.00–23.53)	0.005 *
*Alloprevotella*	1.65 (0.00–4.49)	1.75 (0.00–18.84)	0.068
*Prevotella*	6.45 (0.00–25.95)	1.75 (0.00–15.42)	0.002 *
*Prevotellaceae Ga6A1 group*	0.65 (0.00–3.85)	0.10 (0.00–1.48)	0.006 *
Campylobacterota	2.66 (0.00–21.06)	5.49 (0.00–40.48)	0.940
Campylobacteria	2.66 (0.00–21.06)	5.49 (0.00–40.48)	0.940
Campylobacterales	2.66 (0.00–21.06)	5.49 (0.00–40.48)	0.940
Helicobacteraceae	2.06 (0.00–21–06)	4.10 (0.00–37.48)	0.990
*Helicobacter*	2.06 (0.00–21–06)	4.10 (0.00–37.48)	0.990
Firmicutes	68.24 (47.44–92.90)	68.84 (36.09–98.49)	0.754
Bacilli	16.91 (2.40–56.31)	14.71 (1.33–97.87)	0.774
Erysipelotrichales	5.76 (1.61–12.24)	3.23 (0.00–11.15)	0.019 *
Erysipelatoclostridiaceae	2.43 (0.33–9.68)	1.31 (0.00–7.02)	0.062
*Candidatus* Stoquefichus	0.13 (0.00–0.41)	0.01 (0.00–0.30)	<0.001 *
*Catenibacterium*	1.75 (0.00–9.64)	0.77 (0.00–6.26)	0.557
*Erysipelatoclostridium*	0.35 (0.00–0.92)	0.45 (0.00–4.13)	0.269
*Erysipelotrichaceae UCG–003*	0.20 (0.00–1.00)	0.08 (0.00–1.57)	0.061
Erysipelotrichaceae	3.33 (0.93–6.91)	1.90 (0.00–9.07)	0.011 *
*Allobaculum*	1.70 (0.00–6.73)	0.48 (0.00–3.95)	0.003 *
*Faecalitalea*	0.09 (0.00–0.23)	0.34 (0.00–2.83)	0.535
*Holdemanella*	0.60 (0.00–2.08)	0.92 (0.00–8.93)	0.283
*Turicibacter*	0.43 (0.00–3.04)	0.15 (0.00–1.65)	0.426
Lactobacillales	10.78 (0.01–48.68)	11.14 (0.03–97.13)	0.189
Enterococcaceae	0.10 (0.00–0.92)	4.48 (0.00–96.56)	0.003 *
*Enterococcus*	0.10 (0.00–0.92)	4.48 (0.00–96.56)	0.003 *
Lactobacillaceae	9.50 (0.00–48.45)	1.19 (0.00–17.37)	0.141
*Lactobacillus*	9.50 (0.00–48.45)	1.19 (0.00–17.37)	0.141
Streptococcaceae	1.19 (0.00–10.72)	5.43 (0.00–38.85)	0.021 *
*Streptococcus*	1.18 (0.00–10.72)	5.42 (0.00–38.71)	0.021 *
Staphylococcales	0.01 (0.00–0.04)	0.29 (0.00–5.15)	0.057
Clostridia	43.31 (26.83–66.44)	45.58 (0.43–81.79)	0.754
Clostridiales	1.08 (0.00–4.21)	3.52 (0.02–26.67)	0.643
Clostridiaceae	1.08 (0.00–4.21)	3.52 (0.02–26.67)	0.643
*Clostridium sensu stricto* 1	1.07 (0.00–4.21)	3.40 (0.01–23.97)	0.574
Lachnospirales	21.82 (11.44–37.87)	27.61 (0.19–67.31)	0.402
Lachnospiraceae	21.82 (11.44–37.87)	27.59 (0.19–67.31)	0.416
*Blautia*	12.59 (6.68–22.93)	12.85 (0.00–48.68)	0.476
*Lachnoclostridium*	0.51 (0.00–2.07)	1.99 (0.00–27.09)	0.489
*Lachnospiraceae NK4A136 group*	0.22 (0.00–0.49)	0.13 (0.00–1.38)	0.015 *
*Roseburia*	0.14 (0.00–1.29)	0.88 (0.00–11.61)	0.158
*[Ruminococcus] gnavus group*	0.66 (0.26–9.87)	0.47 (0.02–17.48)	0.767
*[Ruminococcus] torques group*	1.54 (0.00–3.95)	1.16 (0.00–5.94)	0.101
*Sellimonas*	0.27 (0.00–0.73)	0.17 (0.00–2.66)	0.042 *
*Tyzzerella*	0.39 (0.00–1.49)	0.69 (0.00–5.51)	0.521
Oscillospirales	4.40 (0.02–12.67)	2.76 (0.00–15.46)	0.037 *
Butyricicoccaceae	0.35 (0.00–1.30)	0.45 (0.00–7.64)	0.147
*Butyricicoccus*	0.09 (0.00–0.46)	0.13 (0.00–1.74)	0.230
Oscillospiraceae	0.47 (0.00–3.60)	0.01 (0.00–1.60)	0.143
*UCG–005*	0.40 (0.00–2.82)	0.03 (0.00–0.90)	<0.001 *
Ruminococcaceae	3.58 (0.02–8.78)	2.21 (0.00–14.87)	0.070
*Faecalibacterium*	3.15 (0.00–7.82)	1.36 (0.00–11.16)	0.028 *
*Fournierella*	0.18 (0.00–0.73)	0.05 (0.00–0.45)	0.034 *
Peptostreptococcales–Tissierellales	15.69 (3.61–47.54)	11.56 (0.12–19.06)	0.124
Peptostreptococcaceae	15.40 (3.61–47.51)	11.32 (0.12–36.98)	0.143
*Peptoclostridium*	11.10 (0.01–23.95)	9.05 (0.00–33.51)	0.536
*Peptostreptococcus*	0.06 (0.00–0.50)	0.20 (0.00–3.07)	0.216
*Romboutsia*	0.81 (0.00–3.16)	0.72 (0.00–4.48)	0.102
Negativicutes	8.01 (0.004–25.21)	8.55 (0.00–35.98)	0.276
Acidaminococcales	1.64 (0.00–4.34)	0.58 (0.00–9.67)	0.001 *
Acidaminococcaceae	1.64 (0.00–4.34)	0.58 (0.00–9.67)	0.001 *
*Phascolarctobacterium*	1.64 (0.00–4.34)	0.58 (0.00–9.67)	0.001 *
Veillonellales–Selenomonadales	6.37 (0.004–20.87)	7.97 (0.00–35.97)	0.335
Selenomonadaceae	6.36 (0.004–20.87)	7.59 (0.00–35.90)	0.311
*Megamonas*	6.36 (0.004–20.87)	7.59 (0.00–35.90)	0.311
Fusobacteria	9.49 (0.64–21.23)	5.97 (0.00–24.58)	0.052
Fusobacteriia	9.49 (0.64–21.23)	5.97 (0.00–24.58)	0.052
Fusobacteriales	9.49 (0.64–21.23)	5.97 (0.00–24.58)	0.052
Fusobacteriaceae	9.49 (0.64–21.23)	5.97 (0.00–24.58)	0.052
*Fusobacterium*	9.49 (0.64–21.23)	5.97 (0.00–24.58)	0.052
Proteobacteria	2.03 (0.09–8.52)	4.66 (0.02–30.79)	0.311
Gammaproteobacteria	2.03 (0.09–8.52)	4.66 (0.02–30.79)	0.311
Aeromonadales	0.94 (0.00–6.96)	0.11 (0.00–1.61)	0.026 *
Succinivibrionaceae	0.94 (0.00–6.96)	0.11 (0.00–1.61)	0.026 *
*Anaerobiospirillum*	0.80 (0.00–6.96)	0.06 (0.00–1.42)	0.231
*Succinivibrio*	0.15 (0.00–0.78)	0.05 (0.00–1.43)	0.031 *
Burkholderiales	0.72 (0.00–1.54)	0.53 (0.00–3.27)	0.086
Sutterellaceae	0.72 (0.00–1.54)	0.52 (0.00–3.27)	0.075
*Parasutterella*	0.06 (0.00–0.24)	0.02 (0.00–0.21)	0.074
*Sutterella*	0.66 (0.00–1.52)	0.50 (0.00–3.06)	0.275
Enterobacterales	0.36 (0.00–1.96)	3.95 (0.00–30.45)	0.027 *
Enterobacteriaceae	0.24 (0.00–1.95)	3.91 (0.00–30.45)	0.008 *
*Escherichia–Shigella*	0.24 (0.00–1.95)	3.87 (0.00–30.45)	0.011 *

IBD, inflammatory bowel disease; taxa present in at least 50% of the dogs (in any group) were included in this table. * *p*-value < 0.05 using the Wilcoxon signed-rank test.

**Table 6:** Permutational multivariate analysis of variance (PERMANOVA) in bacterial compositions of fecal samples considering different variables.

Variables		Grouping	Pseudo–F	*p*–Value
Clinical condition		HC vs. IBD	4.83	0.006 *
Age		Young (<4)/adult (4–8)/senior (>8)	0.88	0.471
Sex		Male/female	2.13	0.112
Fertile status		Spayed or neutered/entire	1.19	0.312
Breed		Pure-breed/mixed-breed	2.68	0.055
Weight		Small (<10 Kg)/medium-size (10–20 Kg)/large-size (>20 Kg)	2.34	0.048 *
BCS		Low (1–4)/normal (5)/higher (6–9)	1.08	0.398
Living with other pets		Yes/no	3.35	0.031 *
Habitat		Indoor/50–50/outdoor	0.61	0.674
Clinical onset–diagnosis		NA/more than a year/less than a year	2.65	0.037 *
Clinical activity indexes				
CIBDAI [31]		NA, clinically insignificant, mild, moderate, severe	2.07	0.029 *
CCECAI [28]		NA, clinically insignificant, mild, moderate, severe, very severe	1.18	0.316
Endoscopic indexes				
Slovak et al. [32]	Stom. Quan.	Scores (0–4)	1.62	0.111
	Stom. Qual.	Scores (0–3)	1.10	0.353
	Duod. Quan.	Scores (0–6)	0.86	0.598
	Duod. Qual.	Scores (0–4)	0.49	0.893
WSAVA [1]	Esophagus	Scores (0–6)	0.83	0.619
	Stomach	Scores (0–12)	1.20	0.256
	Duodenum	Scores (0–15)	0.79	0.775
Histopathological indexes				
WSAVA [1]	Stomach	Scores (0–8)	0.93	0.559
	Duodenum	Scores (0–18)	1.26	0.180
Abbreviated [33]	Stomach	Scores (0–6)	0.33	0.985
	Duodenum	Scores (0–14)	1.07	0.382

* *p*-value was significant when < 0.05; BCS, body condition score; CIBDAI, canine IBD activity index; CCECAI, canine chronic enteropathy clinical activity index; duod., duodenum; HC, healthy control; IBD, inflammatory bowel disease; NA, not applied; qual., qualitative; quant., quantitative; stom., stomach; WSAVA, World Small Animal Veterinary Association.

## Data Availability

Sequences supporting the conclusions of this article have been deposited into the Sequence Read Archive (SRA) of the National Center for Biotechnology Information (NCBI) under accession number PRJNA905458.

## Supplementary Materials

The following supporting information can be downloaded at: https://www.mdpi.com/article/10.3390/ani13030326/s1, Table S1: Signalment, epidemiological data, and clinical scores of all dogs included in the study.
