# Supplementary material for: Characterization of the Fecal and Mucosa-Associated Microbiota in Dogs with Chronic Inflammatory Enteropathy
Source: Animals (Basel). 2023 Jan 17;13(3):326. doi: 10.3390/ani13030326 (PMC9913788; doi:10.3390/ani13030326)
Supplement: Supplementary file 1 [file animals-13-00326-s001.zip › animals-2104781-supplementary.pdf]

Table S1. Signalment, epidemiological data and clinical scores of all dogs included in the study.

Group	Breed	Age (y)	Sex	Weigh (kg)	BCS	Habitat	Living with other pets	CIBDAI	CCECAI	Sample
HC-1	Jack Russel	5.0	fs	7.5	6/9	Indoor	Yes	0	0	F
HC-2	Mixed breed	1.5	fs	11.7	5/9	Outdoor	Yes	0	0	F
HC-3	Greyhound	2.0	mn	25	6/9	Outdoor	Yes	0	0	F
HC-4	Mixed breed	2.8	f	14	5/9	Indoor	No	0	0	F+D
HC-5	Mixed breed	2.1	mn	13.7	6/9	Indoor	No	0	0	F+D
HC-6	Maltese Bichon	5.0	m	4.5	5/9	Indoor	No	0	0	F+D
HC-7	WHWT	11.0	mn	8.2	7/9	Indoor	No	0	0	F
HC-8	Mixed breed	7.0	fs	4.9	6/9	Outdoor	Yes	0	0	F
HC-9	Greyhound	4.0	mn	22	5/9	Indoor	Yes	0	0	F+D
HC-10	Beagle	9.0	m	16.1	5/9	Indoor	Yes	0	0	F+D
HC-11	English Setter	5.6	fs	15.7	5/9	Indoor	Yes	0	0	F+D
HC-12	Mixed breed	8.8	m	32.8	6/9	Outdoor	No	0	0	F+D
IBD-1	Pyrenean Mountain	2.3	m	44	3/9	50/50	No	7	8	F
IBD-2	Mixed breed	6.9	mn	11.8	5/9	Indoor	No	8	8	F+D
IBD-3	Miniature Schnauzer	3.8	mn	8.4	7/9	Indoor	No	4	6	F
IBD-4	Boston Terrier	3.8	m	10.5	4/9	Indoor	Yes	9	9	F
IBD-5	Mixed breed	12.0	fs	6.4	4/9	Outdoor	Yes	7	10	F
IBD-6	Border Collie	1.9	f	14.6	4/9	Indoor	No	6	7	F+D
IBD-7	Maltese Bichon	11.0	m	4.4	4/9	Indoor	No	4	5	F+D
IBD-8	GSD	4.4	f	29	3/9	50/50	Yes	6	6	F+D
IBD-9	Mixed breed	10.6	fs	6.8	5/9	Indoor	No	6	7	F+D
IBD-10	Maltese Bichon	5.8	fs	4.6	4/9	Indoor	No	8	8	F+D
IBD-11	Mixed breed	3.8	fs	21.4	4/9	Indoor	No	5	7	F+D
IBD-12	Golden Retriever	4.3	fs	23.5	6/9	Indoor	No	3	3	F+D
IBD-13	Yorkshire Terrier	11.0	fs	5	6/9	Indoor	No	7	8	F+D
IBD-14	Mixed breed	3.8	mn	33	5/9	Indoor	No	5	5	F+D
IBD-15	Golden Retriever	2.1	f	36.6	6/9	Indoor	No	6	8	F+D
IBD-16	Bichon Frise	11.8	fs	2.3	5/9	Indoor	No	8	10	F+D
IBD-17	Fox Terrier	11.3	m	8	4/9	Outdoor	No	3	5	F+D
IBD-18	Chihuahua	1.9	fs	3.5	5/9	Indoor	No	8	8	F+D
IBD-19	Maltese Bichon	4.4	fs	3.7	4/9	Indoor	Yes	7	7	F+D
IBD-20	Mixed breed	2.1	f	4.3	4/9	Indoor	No	7	7	F+D
IBD-21	Catalan Shepherd	6.5	fs	17	3/9	Indoor	No	7	9	F+D
IBD-22	GSD	6.5	fs	19.2	2/9	Indoor	Yes	8	10	F+D
IBD-23	Rottweiler	5.4	mn	37.5	4/9	50/50	No	4	5	F+D
IBD-24	French Bulldog	7.1	m	12.4	5/9	Indoor	No	7	9	F+D
IBD-25	Yorkshire Terrier	11.2	m	3.4	3/9	50/50	No	5	7	F+D
IBD-26	Mixed breed	1.5	m	18.4	3/9	Indoor	No	7	7	F+D
IBD-27	Mixed breed	3.9	m	37	4/9	50/50	Yes	6	7	F+D
IBD-28	Beagle	9.5	fs	11.8	4/9	Indoor	Yes	5	6	F+D
IBD-29	Greyhound	10.4	fs	20.8	3/9	50/50	Yes	6	7	F+D
IBD-30	Yorkshire Terrier	5.1	fs	4.5	4/9	Indoor	No	10	12	F+D
IBD-31	Mixed breed	6.7	mn	32.4	6/9	50/50	Yes	6	7	F+D
IBD-32	Mixed breed	2.3	fs	8.7	4/9	Indoor	Yes	6	7	F+D
IBD-33	Pomeranian	8.6	mn	5.7	4/9	Indoor	No	7	8	F+D
IBD-34	Belgian Malinois	1.9	m	27.8	3/9	Indoor	No	8	8	F+D

The canine IBD activity index (CIBDAI) and the canine chronic enteropathy activity index (CCECAI) refers to the clinical activity score at the first visit. BCS, Body condition score; D, duodenal biopsies; F, fecal sample used for microbiota characterization; f, female entire; GSD, German shepherd dog; HC, healthy control; IBD, inflammatory bowel disease; m, male entire; n, neutered; s, spayed; Habitat refers to the area living most part of the day; WHWT, West Highland White Terrier.
